# Receptor-Specific Mechanisms Regulate Phosphorylation of AKT at Ser473: Role of RICTOR in β1 Integrin-Mediated Cell Survival

**DOI:** 10.1371/journal.pone.0032081

**Published:** 2012-02-22

**Authors:** Anjum Riaz, Kathrin Stephanie Zeller, Staffan Johansson

**Affiliations:** Department of Medical Biochemistry and Microbiology, The Biomedical Center, Uppsala University, Uppsala, Sweden; University of Birmingham, United Kingdom

## Abstract

A tight control over AKT/PKB activation is essential for cells, and they realise this in part by regulating the phosphorylation of Ser473 in the “hydrophobic motif” of the AKT carboxy-terminal region. The RICTOR-mTOR complex (TORC2) is a major kinase for AKT Ser473 phosphorylation after stimulation by several growth factors, in a reaction proposed to require p21-activated kinase (PAK) as a scaffold. However, other kinases may catalyse this reaction in stimuli-specific manners. Here we characterised the requirement of RICTOR, ILK, and PAK for AKT Ser473 phosphorylation downstream of selected family members of integrins, G protein-coupled receptors, and tyrosine-kinase receptors and analysed the importance of this phosphorylation site for adhesion-mediated survival. siRNA-mediated knockdown in HeLa and MCF7 cells showed that RICTOR-mTOR was required for phosphorylation of AKT Ser473, and for efficient phosphorylation of the downstream AKT targets FOXO1 Thr24 and BAD Ser136, in response to β1 integrin-stimulation. ILK and PAK1/2 were dispensable for these reactions. RICTOR knockdown increased the number of apoptotic MCF7 cells on β1 integrin ligands up to 2-fold after 24 h in serum-free conditions. β1 integrin-stimulation induced phosphorylation of both AKT1 and AKT2 but markedly preferred AKT2. RICTOR-mTOR was required also for LPA-induced AKT Ser473 phosphorylation in MCF7 cells, but, interestingly, not in HeLa cells. PAK was needed for the AKT Ser473 phosphorylation in response to LPA and PDGF, but not to EGF. These results demonstrate that different receptors utilise different enzyme complexes to phosphorylate AKT at Ser473, and that AKT Ser473 phosphorylation significantly contributes to β1 integrin-mediated anchorage-dependent survival of cells.

## Introduction

Although various stimuli can generate anti-apoptotic signals in cells, most cells are dependent on integrin-mediated adhesion for their survival. Integrins are believed to protect against cell death, mainly by the activation of AKT [1], [2]. Since many growth factor receptors, G protein-coupled receptors, and cytokine receptors also efficiently induce AKT activation [3], the requirement for anchorage is not obvious. Conceivably, it may be due to the more persistent receptor-ligand interactions in focal contacts, or possibly to a different regulation of AKT by integrins.

The activation of AKT family kinases (AKT1–3) involves phosphorylation on Thr308 in the “activation loop” of the catalytic domain and Ser473 in the C-terminal “hydrophobic motif” (the phosphorylation sites are Thr308, Thr309, and Thr305, and Ser473, Ser474, and Ser472 for AKT1–3, respectively; for convenience, these sites are referred to as Thr308 and Ser473 in this article). In addition to these phosphorylation sites required for full AKT kinase activity [4], [5], [6], phosphorylation of other AKT residues also contributes to AKT regulation [7], [8]. The role of phosphatidylinositol-3 kinase (PI3K) in the regulation and activation of AKT became clear when 3-phosphoinositide-dependent protein kinase-1 (PDK1) was identified as the activation loop Thr308 kinase [4], [9]. However, reaching a consensus on the so-called PDK2 (the Ser473 kinase) has proved to be difficult. More than ten different kinases have been proposed to act as PDK2s [10]. The controversy about the physiological PDK2 has mostly revolved around the role of integrin-linked kinase (ILK) [11], DNA-dependent protein kinase (DNA-PK) [12], ataxia telangiectasia mutated (ATM) [13], ataxia telangiectasia and rad-3-related kinase (ATR) [14], protein kinase C (PKC) [15], and RICTOR-mTOR (TORC2) [16].

DNA-PK, ATM and ATR are activated in response to DNA damage, e.g. double-stranded DNA break, and may phosphorylate AKT at Ser473 in such situations [12], [13], [14]. However, growth factor-induced AKT Ser473 phosphorylation does not require DNA-PK, ATM or ATR activity [10], [17], [18]. ILK was, in a number of studies, implicated as a key regulator of the physiological AKT activation. Using different molecular, biochemical and cell-culture approaches, this protein has been shown to bind AKT and to induce phosphorylation at Ser473 [11], [19], [20]. Still, others have seriously questioned the ability of this protein to act as a kinase. ILK lacks some critical structural features considered necessary for kinase activity [21] and its function may be restricted to a role as an adaptor. However, in spite of the atypical active site structure, a Mn^2+^-dependent kinase activity was recently demonstrated for ILK [22], but a comprehensive study by Fukuda et al. [23] has provided evidence against even this kinase function proposed for ILK. Therefore, the role of ILK as a kinase still remains controversial. In contrast to ILK, mTOR in complex with RICTOR [16], [24] and SIN1/MIP1 [25] has been clearly shown to phosphorylate AKT at Ser473. Experiments with human and drosophila cell lines, RICTOR-null mouse fibroblasts, as well as *in vivo* experiments with RICTOR-deficient mice have convincingly shown that TORC2 is a principal physiological PDK2 [16], [26], [27]. However, these studies do not rule out the possibility that other kinase(s) may act as the preferred PDK2 in a context-dependent or cell type-specific manner [10], [28], [29], and phosphorylation of AKT Ser473 was not completely eliminated in RICTOR knockout mouse embryos [26]. Recent studies have suggested that growth factor-induced membrane translocation and activation of AKT requires a protein scaffold provided by p21-activated kinase (PAK) [30]. Others have shown that PAK itself can act as a PDK2 and that inhibition of PAK kinase activity inhibits AKT Ser473 phosphorylation [28], [31], [32].

β1 integrins constitute a main group of cell adhesion receptors to extracellular matrices. Whether ILK, TORC2 or another enzyme is the primary AKT hydrophobic-motif Ser473 kinase specifically downstream of β1 integrins has not been investigated, and this is therefore an important open question. The role of PAK in β1 integrin-induced AKT activation is also unknown. Other central questions regarding the adhesion-dependent survival signalling pathways are: Which AKT isoform(s) is activated by β1 integrins? Is phosphorylation at the hydrophobic motif required for cell survival?

Previously, we have reported differences between β1 integrin- and growth factor-induced PI3K activation [33]. In this study, we investigated the above-mentioned issues of β1 integrin-induced AKT Ser473 phosphorylation. For this purpose, we used siRNAs to knock down ILK, RICTOR-mTOR, or PAK1/PAK2 in HeLa and MCF7 cells and studied the effect of their depletion on the phosphorylation of AKT Ser473 in adhesion assays. To compare the AKT activation-mechanisms utilised by members of major cell surface receptor families in the same cellular context, we also studied the involvement of these proteins in PDGF-, EGF- and LPA-mediated AKT Ser473 phosphorylation. Our results identify similarities as well as stimuli-specific differences in this pathway, and demonstrate the importance of AKT Ser473 phosphorylation for anchorage-dependent survival.

## Results

### β1 integrin-mediated regulation of AKT Ser473 phosphorylation

#### a) RICTOR-mTOR is necessary for AKT Ser473 phosphorylation induced by β1 integrins

**Figure 1:**
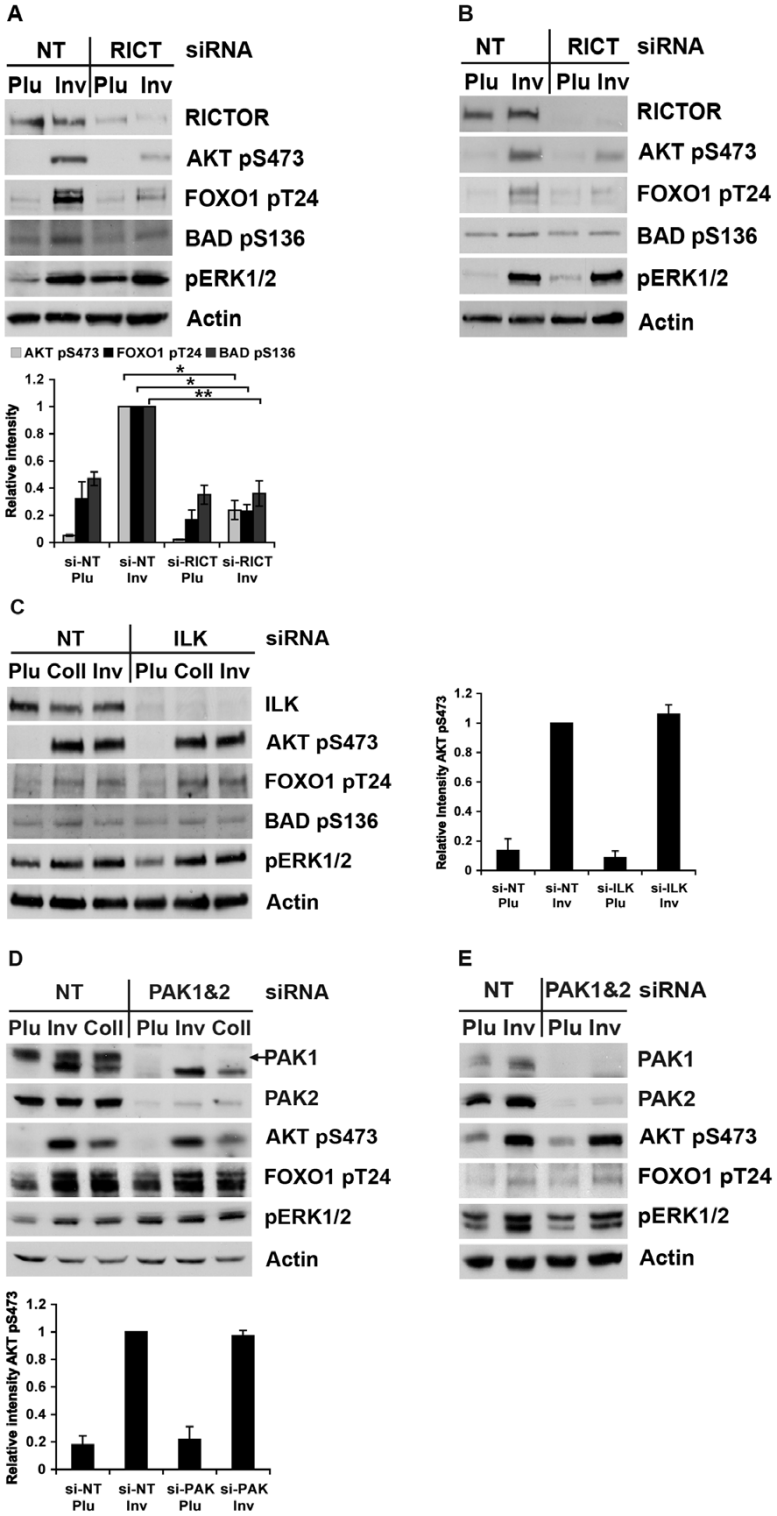
RICTOR knockdown reduces β1 integrin-induced AKT Ser473 phosphorylation but ILK or PAK knockdown has no effect. (A) HeLa cells were transfected with siRNA directed against RICTOR and then allowed to adhere to plates coated with invasin (β1 integrin-ligand) or Pluronic (non-adhesive control) for 60 min. Cell lysates were subjected to SDS-PAGE (4–15% gradient gel) followed by western blotting using the different antibodies as indicated. A representative western blot is shown and quantifications of AKT pSer473, FOXO1 pThr24, and BAD pSer136 on Pluronic and invasin are given below (mean ± s.e.m.; *n* = 3; * represents *p*<0.005 and ** represents *p*<0.05). (B) MCF7 cells were transfected and treated in the same manner as in (A). (C) HeLa cells transfected with ILK-directed siRNA or non-target siRNA were allowed to adhere to plates coated with Pluronic, collagen type I or invasin. Cells were lysed and analysed as explained above. A representative western blot is shown and the graph to the right shows quantification of AKT pSer473 levels on Pluronic and invasin (mean ± s.e.m.; *n* = 3). (D) HeLa cells were transfected simultaneously with PAK1- and PAK2-directed siRNAs or non-target siRNA. Adhesion assays were performed on plates coated with Pluronic, invasin or collagen type I. Cell lysates were subjected to SDS-PAGE (10% gel) followed by western blotting using the different antibodies as indicated. A representative western blot is shown and the graph below presents quantification of AKT pSer473 levels on Pluronic and invasin (mean ± s.e.m.; *n* = 3). (E) Adhesion assay with MCF7 cells transfected with PAK1- and PAK2-directed siRNAs or non-target siRNA, performed as described in (A).

It has been shown that RICTOR-mTOR (TORC2) can directly interact with AKT and phosphorylate Ser473, and it is widely accepted that TORC2 is the principal AKT Ser473 kinase upon growth factor stimulation. To elucidate the role of TORC2 in β1 integrin-induced AKT Ser473 phosphorylation, we used siRNA directed against RICTOR, the rapamycin-insensitive regulatory component of this kinase complex. The effects of RICTOR knockdown in HeLa and MCF7 cells were studied in cell adhesion assays for 60 min on the β1 integrin-binding protein invasin. The RICTOR siRNA efficiently reduced RICTOR protein levels in both cell lines and suppressed integrin-induced AKT phosphorylation at Ser473 (Fig. 1A and 1B). Phosphorylation of the AKT substrate FOXO1 at Thr24 was minimal in HeLa and MCF7 cells seeded in non-adhesive (Pluronic-coated) dishes, and it was strongly stimulated in the control-transfected cells attached to invasin. In cells transfected with RICTOR siRNA, invasin-induced FOXO1 Thr24 phosphorylation was reduced by 72% in HeLa cells, and in MCF7 its level was close to the level seen in non-adherent cells. The β1 integrin-induced phosphorylation of BAD Ser136, an AKT substrate regulating apoptosis, was also reduced after RICTOR knockdown in both cell lines. Interestingly, the BAD Ser136 phosphorylation was maintained at a higher level than FOXO1 Thr24 phosphorylation in non-adherent cells during the assay period (Fig. 1A and 1B). As expected, RICTOR knockdown did not affect β1 integrin-induced ERK1/2 phosphorylation. These data demonstrate that RICTOR-mTOR is the primary PDK2 activated by β1 integrins.

#### b) ILK is not required for β1 integrin-mediated AKT Ser473 phosphorylation

The requirement for ILK in β1 integrin-induced phosphorylation of AKT Ser473 was tested in HeLa and MCF7 cells. As shown in Fig. 1C, transfection of ILK-directed siRNA in HeLa cells resulted in efficient suppression of the protein (Fig. 1C). However, this did not affect the level of β1 integrin-induced AKT phospho-Ser473 (pSer473) during adhesion to invasin or collagen type I. Similar results were obtained for MCF7 cells (data not shown). Thus, while ILK is known to mediate important integrin functions [34], [35], [36], it was dispensable both as a kinase and an adaptor for AKT Ser473 phosphorylation. Consistent with this result, the β1 integrin-induced phosphorylation of FOXO1 Thr24 and BAD Ser136 was not affected by ILK knockdown (Fig. 1C).

#### c) PAK1 and PAK2 suppression does not affect β1 integrin-mediated AKT Ser473 phosphorylation

Group I PAKs (PAK1, PAK2 and PAK3) are of central importance during cell growth and migration [37]. PAK has also been reported to regulate RAC-dependent AKT activation [30], [32], and PAK-induced colon cancer cell survival and invasion require the PI3K-AKT pathway [31]. To investigate whether PAK is needed for β1 integrin-mediated AKT Ser473 phosphorylation or not, we simultaneously knocked down both PAK1 and PAK2 using siRNAs. According to previous studies, knocking down both isoforms is necessary in order to affect cell-signalling cascades involving PAK [38]. Adhesion assays performed with PAK1/PAK2-depleted cells revealed that these proteins were not required for β1 integrin-mediated phosphorylation of AKT Ser473 or of FOXO1 Thr24. Despite efficient knockdown of both PAK1 and PAK2, the adhesion-induced phosphorylation levels at these sites were comparable in non-target siRNA- and PAK1/PAK2 siRNA-transfected cells (Fig. 1D and 1E).

Our attempts to confirm the data obtained from PAK1/PAK2 siRNA experiments using the PAK inhibitor IPA3 were not successful. HeLa and MCF7 cells treated with 20 µM or higher concentrations of IPA3 were unable to spread on invasin. Interestingly, when added to already adherent HeLa and MCF7, IPA3 was well tolerated at concentrations up to 50 µM and cells did not show signs of distress or increased detachment from the surface (data not shown).

#### d) β1 integrins induce Ser473 phosphorylation on both AKT1 and AKT2 isoforms

**Figure 2:**
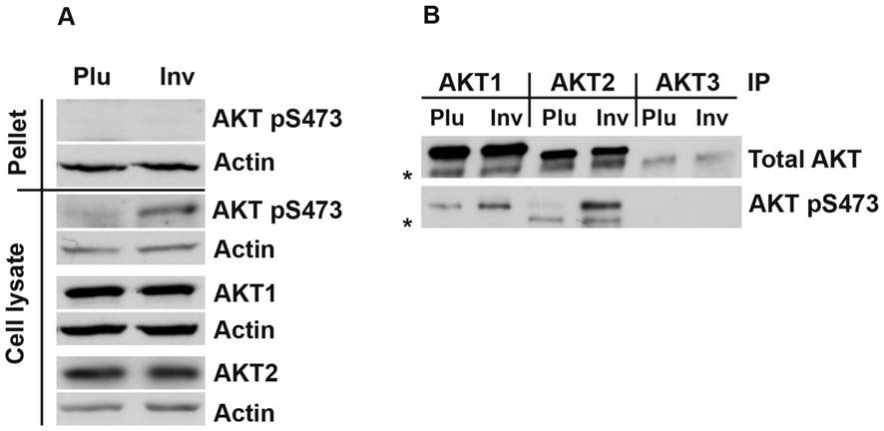
β1 integrins induce Ser473 phosphorylation on both AKT1 and AKT2. Adhesion assays were performed using MCF7 cells and plates coated with Pluronic or invasin followed by immunoprecipitation with isoform-specific AKT1, AKT2 and AKT3 antibodies. (A) The lysates were centrifuged in order to remove the insoluble fraction. The resulting pellet was investigated for the presence of AKT pSer473 by SDS-PAGE and western blotting. A sample of each lysate supernatant after centrifugation was investigated for the presence of AKT pSer473, AKT1 and AKT2 proteins. (B) Immunoprecipitation was performed with antibodies specific for AKT1, AKT2 or AKT3 (included as control) in pre-cleared lysates. The precipitates were boiled and subjected to two parallel SDS-PAGE and western blot analyses in order to detect total AKT or AKT pSer473 protein. In spite of using light-chain specific anti-rabbit-HRP conjugates, some weaker unspecific bands (marked with an *) are visible due to interaction of the primary western antibodies with the heavy chain of the IgGs used for immunoprecipitation.

So far, it is not known whether β1 integrins mediate Ser473 phosphorylation of all AKT family members or just of a certain AKT isoform. A previous study by Dufour et al. [39] reported that suspension culture of Caco-2/15 epithelial cells in serum-free medium for 24 h caused reduced levels of pSer473 on AKT1 and AKT2, and β1 integrin-blocking antibodies could also reduce AKT1 pSer473. In order to more directly elucidate the isoform specificity of β1 integrin-triggered AKT Ser473 phosphorylation, we performed adhesion assays using MCF7 cells on the β1 integrin-specific ligand invasin followed by immunoprecipitation using antibodies for AKT1 and AKT2. AKT3 is not expressed at measurable protein and mRNA levels in MCF7 and HeLa cells [40], and therefore an anti-AKT3 antibody was used as a control. Analysis of the efficiency of the cell lysis conditions used for immunoprecipitation (Triton X-100 based) showed that the pellet fraction obtained after centrifugation of the cell lysate did not contain detectable amounts of AKT pSer473 (Fig. 2A), and thus most of the phosphorylated AKT after β1 integrin-stimulation was present in the soluble fraction (Fig. 2A). While both AKT1 and AKT2 were phosphorylated at Ser473 in response to adhesion, phosphorylation of AKT2 was clearly more induced compared to AKT1 (Fig. 2B).

#### e) Adhesion-dependent signals improve cell survival through RICTOR-mTOR activity

In adhesion-dependent cells, integrin-induced survival signals are required to avoid anoikis [41]. The importance of AKT activity and several of its direct and indirect targets in cell survival is well documented [42], [43]. However, less is known about the contribution of the Ser473 phosphorylation to AKT's pro-survival functions.

**Figure 3:**
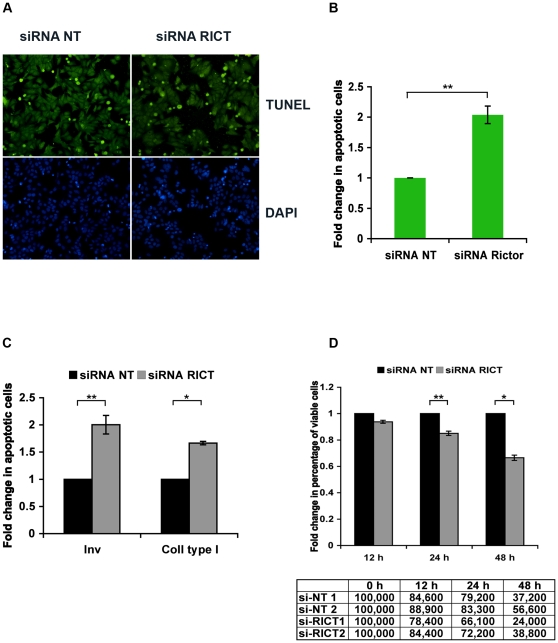
Adhesion-induced RICTOR-mediated AKT Ser473 phosphorylation promotes cell-survival. (A) MCF7 cells transfected with RICTOR-directed or non-target siRNA were grown on glass coverslips in complete culture medium for 48 h, then serum starved for 24 h. A TUNEL assay was performed to visualise apoptotic cells and DAPI was used to stain nuclei. Images shown are representative of pictures taken in three independent experiments. (B) Quantification of (A): Five fields were photographed and at least 400 cells were counted from each coverslip and data is presented as fold change in the percentage of apoptotic cells (mean ± s.e.m.; *n* = 3, ** represents *p*<0.05). (C) MCF7 cells were transfected as described in (A) and after culturing them for 48 h, the cells were trypsinised and 5×10^5^ cells were seeded in starvation medium on invasin- or collagen type I-coated glass coverslips. The cells were incubated in starvation medium for 24 h, fixed, stained with DAPI and observed under a fluorescent microscope. Cells with pyknotic nuclei (condensed and brightly blue-stained) were considered as apoptotic cells. The result is presented as fold change (mean ± s.e.m.; *n* = 2, * represents *p*<0.005 and ** represents *p*<0.05). (D) MCF7 cells were transfected as explained above and subsequently 1×10^5^ cells were seeded onto collagen type I-coated dishes in starvation medium. The cells were trypsinised after the indicated time periods and the number of viable cells was counted using the Trypan blue exclusion method. The plot shows fold change in the percentage of viable cells (mean ± s.e.m.; *n* = 2 and ** represents *p*<0.05) normalised to non-target transfected cells for each time point. The actual numbers of viable cells are given in the table below.

After identification of RICTOR-mTOR as the primary PDK2 downstream of β1 integrins, we investigated the outcome of RICTOR knockdown on integrin-mediated cell survival. Adhesion-mediated protection against apoptosis was determined in MCF7 cells, transfected with non-targeting or RICTOR siRNA, by keeping them in starvation medium. After seeding on glass in complete medium for 48 h, followed by 24 h of serum starvation, the TUNEL assay revealed a 2-fold difference (*p*<0.05) in the number of apoptotic cells between control and RICTOR-depleted cells (9.6% versus 19.6%, Fig. 3A and 3B). To investigate the contribution of β1 integrin-specific ligands to cell survival, non-target or RICTOR-directed siRNA transfected MCF7 cells were seeded in serum-free conditions on glass coated with invasin or collagen type I. In the above-mentioned TUNEL experiments, the presence of pyknotic nuclei, detected by DAPI staining, exactly overlapped with the TUNEL staining (Fig. 3A). Counting of the pyknotic nuclei after DAPI staining revealed that apoptosis was significantly higher in RICTOR siRNA transfected cells on both invasin (2-fold) and collagen type I (1.5-fold; Fig. 3C). To confirm this finding through an alternative approach, the number of viable cells was determined using the Trypan blue exclusion method. After adhesion on collagen type I in serum-free medium, the number of viable cells decreased more rapidly in the RICTOR siRNA transfected cells than in control cells: after 24 h, 69% of the cells were alive in the RICTOR-suppressed cells compared to 81% in the control cells and after 48 h the corresponding values were 31% and 47% (Fig. 3D and E).

To investigate whether Ser473 phosphorylation affected the adhesive functions of integrins, and thereby indirectly caused reduced survival by disturbing other integrin signalling pathways, cell attachment and spreading assays were performed. RICTOR knockdown in HeLa cells had no visible influence on the cell morphology after spreading on invasin, collagen type I or vitronectin (Fig. S1A), and neither did it affect HeLa cell attachment to these integrin ligands after 15 and 30 minutes (Fig. S1B).

### Integrins receptors versus cytokine receptors – common features and differences

#### a) Role of RICTOR-mTOR and PAK in LPA-stimulated AKT Ser473 phosphorylation

**Figure 4:**
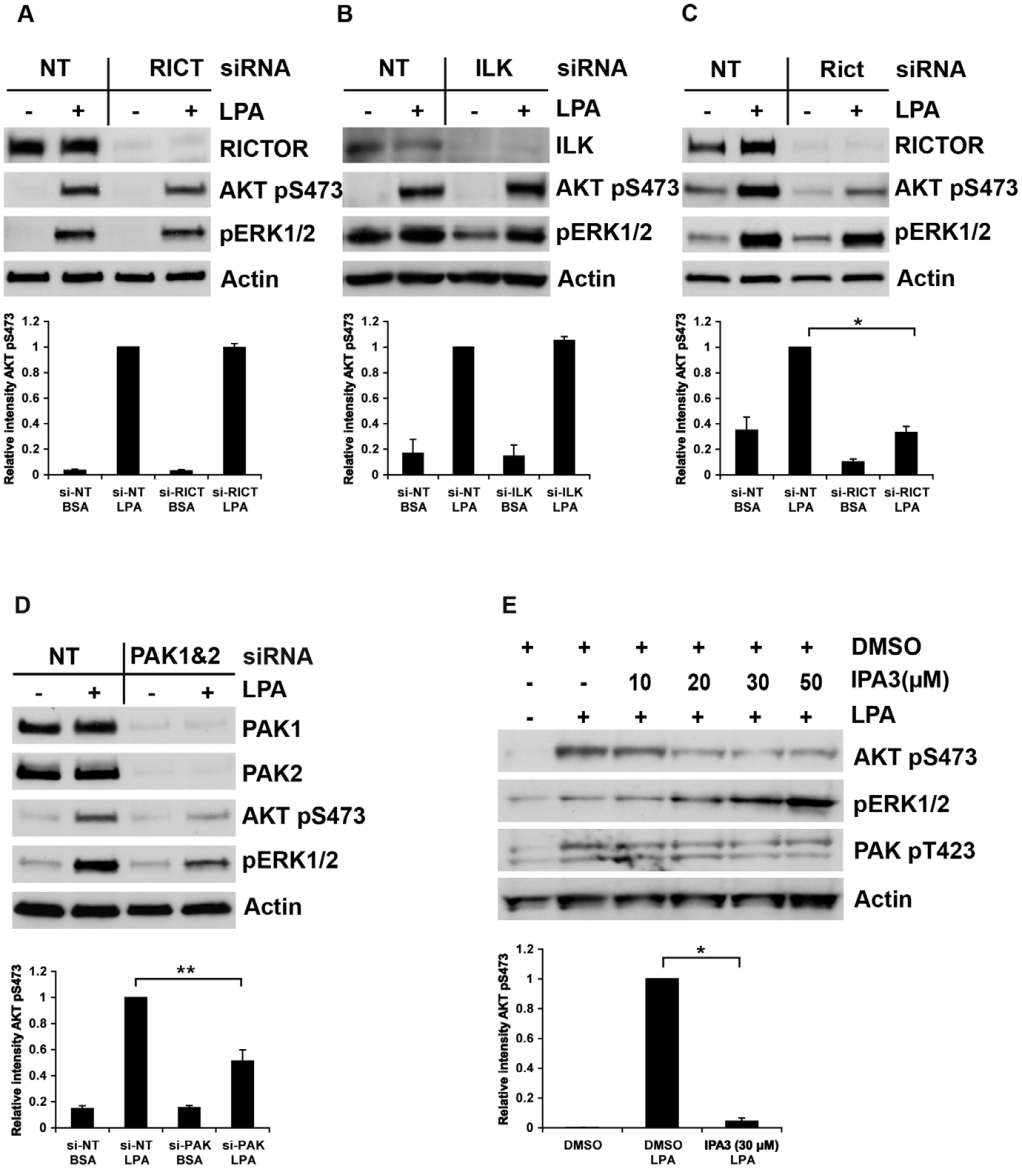
Effects of RICTOR, ILK or PAK knockdown on LPA-induced AKT Ser473 phosphorylation in HeLa and MCF7 cells. (A) HeLa cells were transfected with RICTOR-directed siRNA or non-target siRNA and then stimulated with LPA (10 µM) for 20 minutes. Cell lysates were subjected to SDS-PAGE (4–15% gradient gel) and LPA-induced AKT Ser473 phosphorylation was determined by western blotting. A representative western blot is shown and the graph below provides quantification of AKT pSer473 (mean ± s.e.m.; *n* = 3). (B) HeLa cells transfected with ILK-directed siRNA and stimulated by LPA were analysed as described above. The graph below shows quantification of AKT pSer473 (mean ± s.e.m.; *n* = 2). (C) MCF7 cells transfected as indicated were stimulated with LPA (5 µM) for 5 min and analysed as described above. A representative western blot is presented and the graph below shows quantified levels of AKT pSer473 (mean ± s.e.m.; *n* = 3, * represents *p*<0.005). (D) MCF7 cells were transfected simultaneously with PAK1 and PAK2 siRNA or with non-target siRNA and then stimulated with LPA (5 µM) for 5 min. A representative western blot is shown. The graph below is a quantification of AKT pSer473 levels (mean ± s.e.m.; *n* = 3, ** represents *p*<0.05). (E) HeLa cells were treated with increasing concentrations of the PAK inhibitor IPA3 or with DMSO and then stimulated with LPA (10 µM) for 20 min. The graph below is the quantification of AKT pSer473 levels after LPA-stimulation in cells treated with IPA3 (30 µM) normalised to the pSer473 level of this protein in LPA-stimulated cells without the inhibitor (mean ± s.e.m.; *n* = 3, * represents *p*<0.005).

Analysis of the AKT Ser473 phosphorylation mechanism after LPA stimulation revealed differences from the integrins pathway. First, RICTOR knockdown did not affect the AKT Ser473 phosphorylation in response to LPA in HeLa cells (Fig. 4A), and neither did knockdown of ILK (Fig. 4B). This result was confirmed by knocking down RICTOR and ILK simultaneously (Fig. S2). In contrast, in MCF7 cells, RICTOR suppression markedly reduced LPA-mediated phosphorylation of AKT at Ser473 (Fig. 4C). In both the cell lines, LPA-induced ERK1/2 phosphorylation remained essentially unaffected. HeLa and MCF7 cells also exhibited different kinetics in the response to LPA: MCF7 cells gave maximum response when stimulated with 5 µM LPA for 3–5 min (Fig. S3A), while HeLa cells required LPA stimulation for 20 min (Fig. S3B) to obtain detectable AKT Ser473 phosphorylation. Since HeLa and MCF7 cells express different LPA receptors [44], [45], our results suggest that distinct members of this G protein-coupled receptor family use different mechanisms for AKT activation.

Second, in contrast to β1 integrin-induced AKT Ser473 phosphorylation, the LPA-stimulated phosphorylation was decreased in MCF7 cells treated with PAK1/PAK2 siRNA (Fig. 4D). This result was confirmed with the PAK inhibitor IPA3 in HeLa cells as shown in Fig. 4E. A reduction in AKT pSer473 was seen in cells treated with 20 µM IPA3, and no further effect was obtained at higher inhibitor concentrations. IPA3 treatment did not reduce phosphorylation of PAK itself at Thr423/402. These data are in agreement with the proposed model of PAK inhibition by IPA3, where IPA3 stabilizes PAK in an inactive conformation that prevents autophosphorylation at Thr423, but it does not inhibit the phosphorylation of this site by exogenous kinases [46]. Interestingly, ERK1/2 phosphorylation in response to LPA increased in cells treated with IPA3. This increase was IPA3 dose-dependent (Fig. 4E). A similar effect of IPA3 on basal ERK1/2 phosphorylation has previously been observed [46].

#### b) PDGF and EGF receptors use different routes to AKT Ser473 phosphorylation

**Figure 5:**
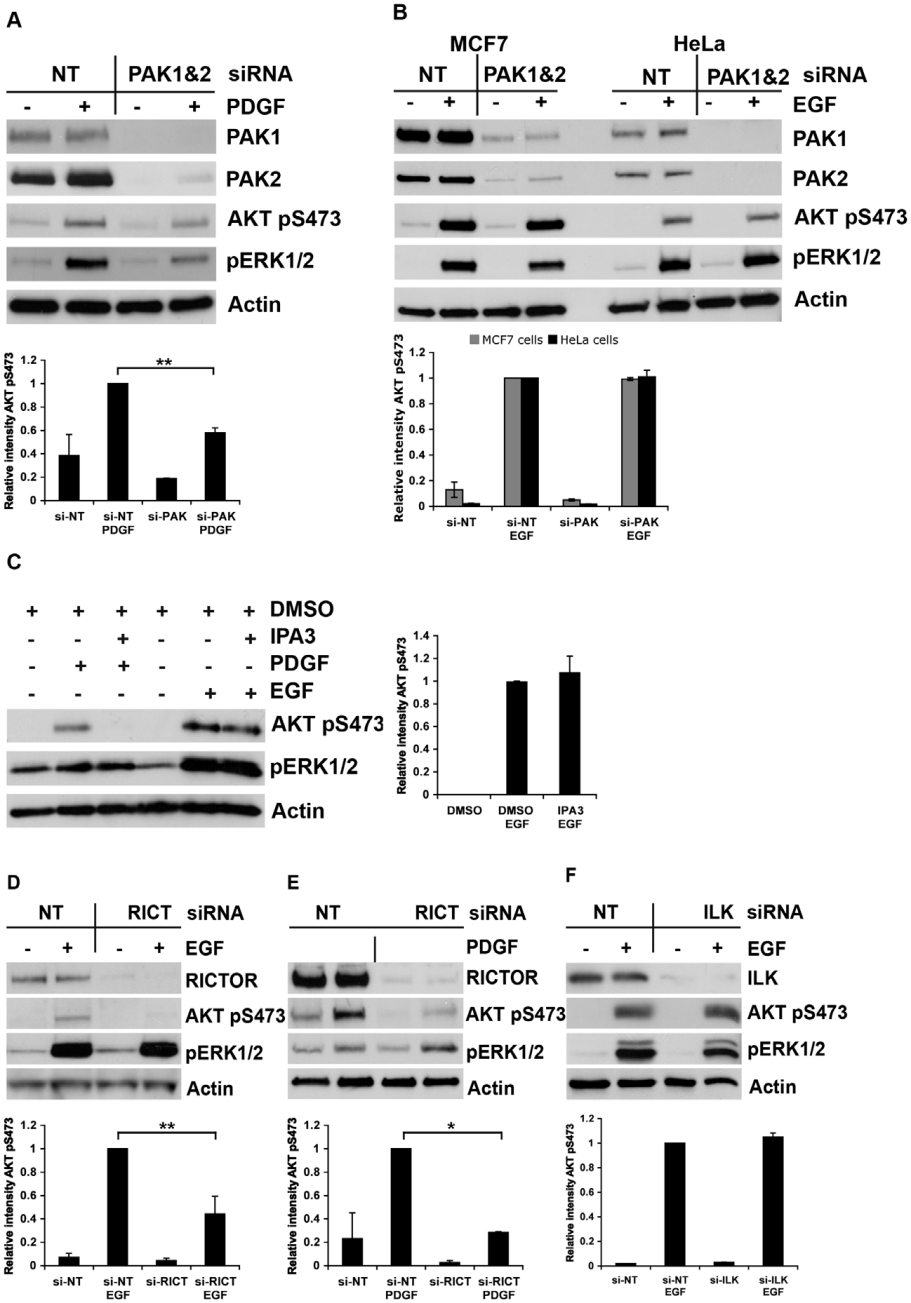
PAK is necessary for PDGF but not for EGF-mediated AKT Ser473 phosphorylation whereas RICTOR knockdown inhibits both pathways. (A) PAK1 and PAK2 expression was suppressed in MCF7 cells using siRNAs and the cells were stimulated with 20 ng/ml PDGF-BB for 10 min. A representative western blot is shown and AKT pSer473 is quantified below (mean ± s.e.m.; *n* = 2, ** represents *p*<0.05). (B) MCF7 and HeLa cells were transfected as above and stimulated with 20 ng/ml EGF. A representative western blot is shown and below quantification of AKT pSer473 in MCF7 and HeLa cells (mean ± s.e.m.; *n* = 3) is presented. (C) HeLa cells were treated with PAK inhibitor IPA3 (30 µM) or DMSO as vehicle control and then stimulated with 20 ng/ml PDGF-BB or EGF. A representative western blot is shown. The graph provides quantification of AKT pSer473 levels, after EGF-stimulation of cells treated with IPA3 (30 µM) normalised to the pSer473 level of this protein in EGF-stimulated cells without the inhibitor (mean ± s.e.m.; *n* = 3). (D) RICTOR expression was suppressed in HeLa cells using siRNA and the cells were stimulated with EGF (20 ng/ml). Cell lysates were subjected to SDS-PAGE followed by western blotting using antibodies as indicated. A representative blot is shown and quantification of AKT pS473 levels is found below (mean ± s.e.m.; *n* = 3, ** represents *p*<0.05). (E) HeLa cells transfected with RICTOR-directed siRNA were stimulated with 20 ng/ml PDGF-BB and analysed as in (D). A representative western blot is shown and the graph below shows quantification of AKT pSer473 (mean ± s.e.m.; *n* = 2, * represents *p*<0.005). (F) HeLa cells, transfected with non-target or ILK-directed siRNA, were stimulated with 20 ng/ml EGF and the samples analysed as explained above. The graph shows quantification of AKT pSer473 (mean ± s.e.m.; *n* = 2).

Differences in the requirement for PAK were found not only between β1 integrins and LPA receptors, but also among growth factor receptors. PAK1/PAK2 knockdown reduced PDGF-stimulated AKT phosphorylation at Ser473 in MCF7 cells (Fig. 5A) as previously reported for COS1 and NIH3T3 cells [30]. In contrast, it did not inhibit EGF-induced AKT pSer473 in either MCF7 or HeLa cells (Fig. 5B). This difference in PAK-dependency was not caused by the different kinetics of Ser473 phosphorylation induced by PDGF or EGF because it was present during the entire response period (Fig. S4A and 4B). Consistent with the siRNA data, IPA3 inhibited induction of AKT Ser473 phosphorylation by PDGF but not by EGF (Fig. 5C).

Other features of the AKT Ser473 phosphorylation pathway were similar for PDGF and EGF receptors as for β1 integrins: RICTOR knockdown reduced AKT Ser473 phosphorylation (Fig. 5D, E), and this phosphorylation event occurred independently of ILK (Fig. 5F).

## Discussion

Regulation of AKT activity is a tightly controlled event and its importance is evident from the pathological states that emerge due to the loss of this control. In particular, elevated levels of AKT activity are linked to tumour progression and resistance to anti-cancer treatments [47], [48]. As an important part of AKT regulation, several phosphorylation sites on AKT have evolved. Most studied is the phosphorylation of Thr308 and Ser473, both reactions being strongly induced by several types of stimuli and used as markers for AKT activation. However, while phosphorylation of Thr308 is strictly required for AKT kinase activity, the role of pSer473 is still not fully understood. Three major functions have been proposed for the latter phosphorylation site: a) It has been reported to strongly promote binding of PDK1 to AKT, and thereby to facilitate the subsequent activating phosphorylation of Thr308 [16], [49]. b) The serine-phosphorylated hydrophobic motif may also interact intra-molecularly with the catalytic domain of AKT and stabilize its activated state [50]. c) It may affect the substrate specificity of AKT [25]. Notably, none of these functions exclude the others.

Both PDK1 and PDK2 are dependent on 3′-phosphorylated membrane phosphatidylinositols for phosphorylation of AKT, and we previously showed that β1 integrins use the p110α PI3K catalytic subunit for the generation of such phospholipids [51]. Our primary aims for the present investigation were to clarify the mechanism of AKT Ser473 phosphorylation during adhesion via β1 integrins and to determine its contribution to adhesion-mediated survival of cells. We used two human carcinoma cell lines, the epithelial-like MCF7 and the mesenchymal-like HeLa, which can efficiently be transfected with siRNA.

siRNA-mediated knockdown of the main PDK2 candidates, TORC2 and ILK, revealed that TORC2, but not ILK, was necessary for AKT Ser473 phosphorylation downstream of β1 integrins (Fig. 1A–C). Our experiments thus extend the existing evidence about the RICTOR-mTOR complex as the principal PDK2 downstream of growth factor receptors to also include β1 integrins. ILK has been shown to associate with β1 integrins [34] and a large body of literature directly or indirectly implicates ILK in AKT regulation and as a PDK2 [11], [20], [35], [52], [53]. However, convincing evidence provided by other studies contradicts claims made about ILK's kinase functions [21], [23] and concludes that it performs important tasks as an accessory protein in several signalling events. Depletion of ILK by siRNA or conditional knockout were reported to cause reduced AKT Ser473 phosphorylation in various cell lines, including MCF7 [20], [52]. In contrast, using siRNA-mediated knockdown, we could not establish a function for ILK in β1 integrin-, EGF- or LPA-mediated AKT Ser473 phosphorylation in HeLa and MCF7 cells. These seemingly conflicting results most likely reflect the different assay conditions used. In the previous studies, the cells were harvested and analysed after several days of continuous growth in serum without any specific stimuli, while acute stimulation of selected receptors was used in the present study. Since ILK participates in the regulation of basic cell functions such as focal contact assembly and actin dynamics [35], [36], the effects of ILK depletion on AKT phosphorylation during long term experiments may conceivably be indirect rather than targeting the actual AKT phosphorylation mechanism. However, at present the possibility cannot be excluded that ILK may have a scaffold function for AKT activation downstream of a particular stimuli in a certain cell type, but this remains to be demonstrated.

Immunoprecipitation with isoform-specific antibodies showed that both AKT1 and AKT2 were phosphorylated on Ser473 in response to adhesion. Notably, while the Ser473 phosphorylated AKT1 and AKT2 migrated equally fast in SDS-PAGE, the main pool of AKT2 migrated slightly faster than AKT1 (Fig. 2B). This observation was made in three different cell lines, MCF7 (Fig. 2B), HeLa and GD25 (data not shown) using two different AKT2 antibodies (Abcam, ab66129 and Santa Cruz, sc-81436), suggesting that the shift in mobility, possibly caused by additional modifications of AKT2, is commonly occurring during its activation. AKT Ser473 phosphorylation was more pronounced for AKT2 than AKT1 during spreading of MCF7 cells on invasin, although AKT1 seemed more abundant in the cells (Fig. 2B). The prominent activation of AKT2 by β1 integrins is particularly interesting in view of its reported localization to mitochondria in MCF7 cells as well as several other cell lines [40]. Furthermore, AKT2 was found to prevent autophagy of mitochondria and to have a major role in protection against apoptosis [54]. It is presently unknown whether AKT2 is activated at the plasma membrane and subsequently translocated to the mitochondria or if integrins can directly activate AKT2 residing on the mitochondria.

In adhesion-dependent cells, integrins promote cell survival mainly through the PI3K-AKT pathway [1], [2]. It is well established that blocking integrin signals makes cells vulnerable to apoptosis. Furthermore, Uesugi et al. [55] have recently identified a microRNA, miR-218, that inhibits AKT Ser473 phosphorylation by reducing RICTOR protein levels. Their findings that forced miRNA-218 expression in oral cancer cell lines, lacking endogenous miRNA-218, can induce caspase-mediated apoptosis in these cells, highlights the relevance of RICTOR-regulated AKT activity to apoptosis protection. Our studies demonstrated that RICTOR-dependent AKT Ser473 phosphorylation aids β1 integrins in protecting cells from cell death under serum starvation conditions. This effect can at least partly be ascribed to activation of the important anti-apoptotic AKT target BAD, since β1 integrin-induced phosphorylation of BAD Ser136 was reduced after RICTOR knockdown (Fig. 1A and 1B). Phosphorylation of BAD Ser136 prevents BAD from binding to Bcl2, and thereby allows Bcl2 to inactivate the pro-apoptotic BAX [56]. However, BAD pSer136 levels were relatively high in HeLa and MCF7 cells after RICTOR knockdown compared to the level of FOXO1 pThr24 (Fig. 1A and 1B). Phosphorylation of FOXO1 Thr24 and a few other unidentified AKT substrates were previously shown to be strongly dependent on TORC2-mediated AKT phosphorylation at Ser473, while several other AKT substrates were less affected by disruption of the TORC2 complex [25]. Our data indicates that BAD belongs to the latter group of AKT targets, and that the reduced BAD phosphorylation after RICTOR knockdown reflects a general lower AKT activity rather than involvement of phosphorylated Ser473 in recognition of BAD as substrate. Another difference between BAD and FOXO was the complete dephosphorylation of FOXO in non-adherent cells within 2 hours (the total time the cells remained in suspension in our adhesion assay, as explained in the methods section), while a higher level of phosphorylated BAD was retained. Possibly, the different turnover kinetics are due to the interaction of 14-3-3 proteins with BAD pSer136 [56].

LPA receptors, and several other members of the G protein-coupled receptor family, can also activate the PI3K-AKT pathway [57]. This activation involves the action of Gi, PI3K p110β and PDK1 [58]. Our attempts to study the possible role of TORC2 downstream of LPA produced diverging results. In HeLa cells, RICTOR knockdown, and even simultaneous RICTOR and ILK knockdown, did not reduce LPA-generated AKT Ser473 phosphorylation (Fig. 4A and Fig. S2). In contrast, RICTOR suppression did reduce Ser473 phosphorylation in MCF7 cells (Fig. 4C). MCF7 cells mainly express the receptor LPA2 and some LPA1 [45], while HeLa cells express receptor LPA3 in addition to a lower level of LPA2 [44]. Possibly, these differences in the available LPA receptors resulted in different responses after RICTOR suppression, i.e. LPA3 may activate another AKT hydrophobic-motif kinase. This reasoning is supported by our observations that HeLa cells respond to LPA with much slower kinetics (Fig. S3A), compared to MCF7 cells (Fig. S3B). However, our data is insufficient to unambiguously connect the upstream individual LPA receptors with specific PDK2s preferred for AKT activation. Nevertheless, our results are consistent with the results by Shiota et al. [26], which showed that alternative PDK2s exist beside TORC2 since residual Ser473-phosphorylated AKT was detected in RICTOR knockout mouse embryos.

A recent report by Higuchi et al. [30] has brought PAK's role in AKT activation into focus. They provide evidence that PAK is involved in the PI3K-AKT pathway as a scaffolding protein. Other studies also suggest a need for PAK in AKT activation [28]. However, in our adhesion assays, PAK1/PAK2 knockdown did not reduce β1 integrin-induced phosphorylation of AKT Ser473 (Fig. 1D and 1E). These results are not in conflict with the findings of Higuchi et al. [30] since their work focussed on the role of PAK in growth factor-induced AKT activation. The results of our experiments with PDGF were consistent with their data (Fig. 5A).

Another case of PAK-independent AKT Ser473 phosphorylation was observed after EGF-stimulation in MCF7 and HeLa cells (Fig. 5B and 5C). Nakamura et al. [59] have recently shown that the EGF receptor uses Freud1/Aki1 as a PI3K and PDK1 scaffolding protein. However, Freud1 inhibition only reduced phosphorylation at Thr308 and had no effect on Ser473. Thus, while both PAK and Freud1/Aki1 are dispensable for AKT Ser473 phosphorylation downstream of the EGF receptor, these examples indicate that the use of scaffold proteins may be a common feature in AKT activation by different receptors.

To conclude, we have provided evidence for a role of TORC2 as the PDK2 activated by β1 integrins, and that Ser473 phosphorylation of AKT1/2 significantly contributes to adhesion-induced survival signals. Our data also highlights the involvement of PAK in LPA and PDGF pathways to Ser473 phosphorylation and indicates the need for further in-depth studies to elucidate the role of PAK, and possibly other scaffold proteins, in AKT activation. A general finding of our study is that the important phosphorylation reaction of AKT Ser473 is more complex than depicted in current models and that the mechanism varies depending on the stimulated receptor.

## Materials and Methods

### Cells

HeLa and MCF7 cells were cultured in Dulbecco's modified Eagle's medium (DMEM, GIBCO Invitrogen) containing 10% fetal bovine serum, penicillin-streptomycin and fungizone. All experiments were performed with cells in log-phase growth.

### Antibodies, siRNAs and other reagents

The following antibodies used in this study were from Cell Signaling Technology, USA: anti-AKT pSer473 (#9271), anti-pERK1/2 (#9106), anti-PAK1(pThr423)/PAK2(pThr402) (#2601), anti-PAK1 (#2602), anti-PAK2 (#2608), anti-AKT3 (#3788), anti-total AKT (#9272), anti-FOXO1 pThr24/FOXO3a pThr32 (#9464), anti-BAD pS136 (#4366). AKT1 antibody (ab6076) was bought from Abcam, anti-RICTOR (A300-459A) from Bethyl, USA, and anti-AKT2 (sc-81436), anti-ILK (sc-7516), HRP-conjugated anti-actin (sc-1616) and HRP-conjugated donkey anti-goat secondary antibody (sc-2020) were from Santa Cruz Biotechnologies. HRP-conjugated donkey anti-rabbit (NA9340) and HRP-conjugated sheep anti-mouse (NA9340) were from GE Healthcare, UK. Light-chain specific mouse-anti-rabbit peroxidase (211-032-171) was from Jackson Immuno Research, Protein A/G PLUS agarose (sc-2003) from Santa Cruz and Sepharose 4B from Pharmacia. The ECL kit (#RPM2106) was from GE Healthcare, Uppsala. The siRNAs against RICTOR, ILK, PAK1, PAK2 and a non-target siRNA were from Thermo Scientific (Dharmacon, siGENOME SMARTpool). All siRNAs were diluted in siRNA buffer (Dharmacon) to 20 µM stock solutions, which were aliquoted and stored at −20°C. The siRNA transfections were performed using Ingenio™ electroporation solution (MirusBio, USA) and AMAXA Nucleofector® (Lonza, USA). Group-I PAK inhibitor IPA3, fatty acid-free BSA and lysophosphatidic acid (LPA) sodium salt were purchased from Sigma. IPA3 stock solution (14 mM) was prepared in cell culture grade DMSO (Sigma). LPA stock solution (2.3 mM) was prepared in chloroform∶methanol∶acetic acid (95∶5∶5). Bovine dermal collagen type I was from Cellon, Luxembourg. The Click-iT® TUNEL assay kit (#C10245) was obtained from Invitrogen.

### siRNA transfections

Two days before transfection, the cells were sub-cultured in order to obtain around 80% confluence on the day of transfection. The cells were washed with PBS and detached from the flask surface by trypsin-EDTA treatment for 15 min at 37°C. Trypsin was inactivated by serum-supplemented medium and the cells were counted. The optimised transfection protocols for HeLa and MCF7 cell lines recommended by Lonza were used. Briefly, each transfection was performed with 5×10^5^ HeLa and 2×10^6^ MCF7 cells. The respective cell pellet was re-suspended in 100 µl of Ingenio™ transfection reagent and the required siRNA against RICTOR, ILK, PAK1 or PAK2 was mixed with the cell suspension. A control reaction using non-target siRNA was also included. The transfected cells were allowed to grow for 48–72 h before further experiments.

### Adhesion assay

Signal transduction reactions generated by β1 integrins were studied in cell adhesion experiments on invasin-, collagen type I- or Pluronic-coated wells of six-well cell culture plates as reported previously [33]. Briefly, after coating with integrin ligands, any remaining uncoated plastic surface was blocked with Pluronic. Cells were serum-starved over-night and then harvested with trypsin-EDTA. Trypsin was inactivated with soybean trypsin inhibitor; the cells were washed and then allowed to rest in suspension for one h at 37°C. Subsequently, they were seeded and allowed to adhere for 60 min at 37°C. The plates were put on ice and the medium including non-attached cells was collected and centrifuged. The resulting cell pellet was then combined with the lysate from adherent cells of the corresponding well, which was harvested by scraping the well with SDS-PAGE sample buffer (SDS-SB). The lysates were analysed by SDS-PAGE followed by western blotting.

### Immunoprecipitation

MCF7 cells were serum-starved over-night and adhesion assays were performed in tissue culture plates (10 cm diameter) coated with invasin or Pluronic as described above. One h after seeding the cells, the non-attached cells were collected and centrifuged at 4°C. In the meanwhile, Triton-lysis buffer (1×TBS, Roche complete Mini protease inhibitor cocktail, 1% Triton X-100, 200 µM Na_3_VO_4_, 125 µM NaF) was added to the plates on ice and the adherent cells were lysed and scraped. These lysates were combined with the corresponding pellets after the centrifugation and then incubated end-over-end in the cold for 20 min. “Total lysate” samples were taken out and boiled with SDS-SB before the remaining lysate was centrifuged at 16000× g for 10 min at 4°C to remove Triton-insoluble material. The resulting supernatant was pre-cleared with a mixture of Sepharose 4B and protein A/G agarose for at least 30 min at 4°C and the pellets were boiled with SDS-SB as “pellet control”. After the pre-clearing, AKT isoform-specific antibodies were added according to the manufacturer's recommendation and incubated end-over-end for one h. This was followed by one h incubation with protein A/G agarose end-over-end at 4°C. Finally, the agarose pellets were washed three times with wash buffer (PBS; 0.1% Triton X-100, Roche Mini protease inhibitor cocktail, 200 µM Na_3_VO_4_, 125 µM NaF) and then boiled in SDS-SB. During the subsequent western blot analysis, light-chain specific anti-rabbit HRP secondary antibodies were used to avoid disturbance by the primary IgG heavy chains.

### PDGF-BB-, EGF- and LPA-stimulation

All the experiments involving PDGF-BB-, EGF- and LPA- stimulation of cells were performed on cells grown in a six-well cell culture plate. Prior to stimulation, the cells were starved in serum-free DMEM for 24 h. PDGF-BB and EGF stimulation reactions were carried out for 10 min using 20 ng/ml of each growth factor. For LPA stimulation, the required volume of LPA was transferred to an Eppendorf tube and the solvent (chloroform, methanol, acetic acid) was evaporated under nitrogen gas. LPA was re-constituted in PBS containing 1 mg/ml fatty acid-free BSA and added to the cells kept in fatty acid-free BSA-containing starvation medium. HeLa and MCF7 cells were stimulated with 10 mM and 5 mM LPA, respectively, in order to obtain maximal responses.

### Inhibitor treatment

The stock solution of PAK inhibitor IPA3 was prepared as described under reagents. The desired working dilution of this inhibitor was prepared directly in starvation medium. Equally diluted DMSO was used as control. Starved cells (24 h) were treated with either the inhibitor or DMSO for 30 min and then used for further experiments.

### SDS electrophoresis and western blotting

Proteins were resolved on pre-cast polyacrylamide mini gels (BioRad) after reduction with 50 mM DTT and then transferred to Hybond-ECL nitrocellulose membrane (GE Healthcare, UK). The membranes were blocked for one h in 5% milk in TBS containing 0.1% Tween 20 (TBS-T). All primary antibody incubations were carried out at 4°C over-night. All secondary antibody incubations were performed at room temperature for one h. The membranes were incubated with ECL substrate, and the signals were detected using x-ray films.

### Determination of apoptosis using Trypan blue exclusion, TUNEL and DAPI staining

Cell viability during serum starvation was determined in cells transfected with siRNA against RICTOR and non-target siRNA, respectively. After transfection, the cells were grown for 48 h followed by trypsin-EDTA treatment and seeding onto collagen-coated dishes at a density of 1×10^5^ cells per dish in starvation medium. Subsequently, cell viability was determined after 12, 24 and 48 h by the Trypan blue exclusion method.

Serum starvation-induced apoptosis was determined using TUNEL and DAPI staining. Cells transfected as described above were grown on glass cover slips for 48 h in complete medium and then incubated in serum-free starvation medium for 24 h. DNA breaks were detected using a TUNEL assay kit (Invitrogen) according to the manufacturer's protocol. Slides were viewed under a fluorescent microscope (Nikon, ECLIPSE 90i) and bright green coloured cells were considered as apoptotic cells. Alternatively, MCF7 cells were transfected and grown in complete culture medium for 48 h before they were trypsinised, and 500,000 cells were seeded onto glass cover slips coated with invasin (20 µg/ml) or collagen type I (50 µg/ml). After incubation in starvation medium for 24 h the cells were fixed with methanol, stained with DAPI, and observed under a fluorescent microscope. Cells with condensed, brightly blue-stained pyknotic nuclei were considered as apoptotic cells.

### Statistical analysis

Student *t*-test was used to determine statistical importance of the data. A *p* value <0.05 was considered statistically significant, and in the figures one asterisk (*) represents *p*<0.005 and ** represents *p*<0.05.

## Supporting Information

Figure S1 **(A) Morphology of HeLa cells transfected with either non-target or RICTOR siRNA after 30 min of spreading on the indicated substrates at 37°C.** (B) Attachment assay with HeLa cells transfected with either non-target or RICTOR siRNA. The cells were essentially treated as described under the adhesion assay heading in the Materials and Methods section. 20,000 serum-starved cells per well were seeded in invasin-, collagen-, vitronectin- or Pluronic-coated wells of a 96-well plate and allowed to attach for 15 or 30 min at 37°C. Non-attached cells were removed and the remaining cells were fixed with 96% ethanol for 10 min. Cells were stained for 20 min with 0.1% crystal violet and solubilised in 0.5% SDS. Absorbance was measured at 600 nm.
(TIF)

Figure S2 **Simultaneous knockdown of RICTOR and ILK does not reduce LPA-mediated AKT Ser473 phosphorylation in HeLa cells.** The cells were transfected with RICTOR- and ILK-directed siRNAs simultaneously or with non-target control siRNA and then stimulated with LPA (10 µM) for 20 min. Knockdown efficiency of RICTOR and ILK, and the phosphorylation status of AKT at Ser473 were analysed by western blotting.
(TIF)

Figure S3 **The kinetics of LPA-induced AKT Ser473 phosphorylation are different in HeLa and MCF7 cells** (A) HeLa cells were serum-starved and stimulated with LPA (10 µM) for different time periods as indicated. (B) Serum-starved MCF7 cells were stimulated with LPA (5 µM).
(TIF)

Figure S4 **PAK knockdown inhibits PDGF-mediated AKT pSer473, but does not affect kinetics of EGF-induced AKT pSer473.** MCF7 cells were transfected simultaneously with PAK1- and PAK2-directed siRNAs or non-target siRNA, grown in complete culture medium for 48 h, serum-starved for 24 h and then stimulated with 20 ng/ml PDGF-BB (A) or 20 ng/ml EGF (B) for the indicated time periods. The efficiency of PAK1 and PAK2 knockdown and the effect of PAK suppression on AKT pSer473 were analysed by western blot.
(TIF)
