# Supplementary figures and images for: Receptor-Specific Mechanisms Regulate Phosphorylation of AKT at Ser473: Role of RICTOR in β1 Integrin-Mediated Cell Survival
Source: PLoS One. 2012 Feb 22;7(2):e32081. doi: 10.1371/journal.pone.0032081 (PMC3284553; doi:10.1371/journal.pone.0032081)

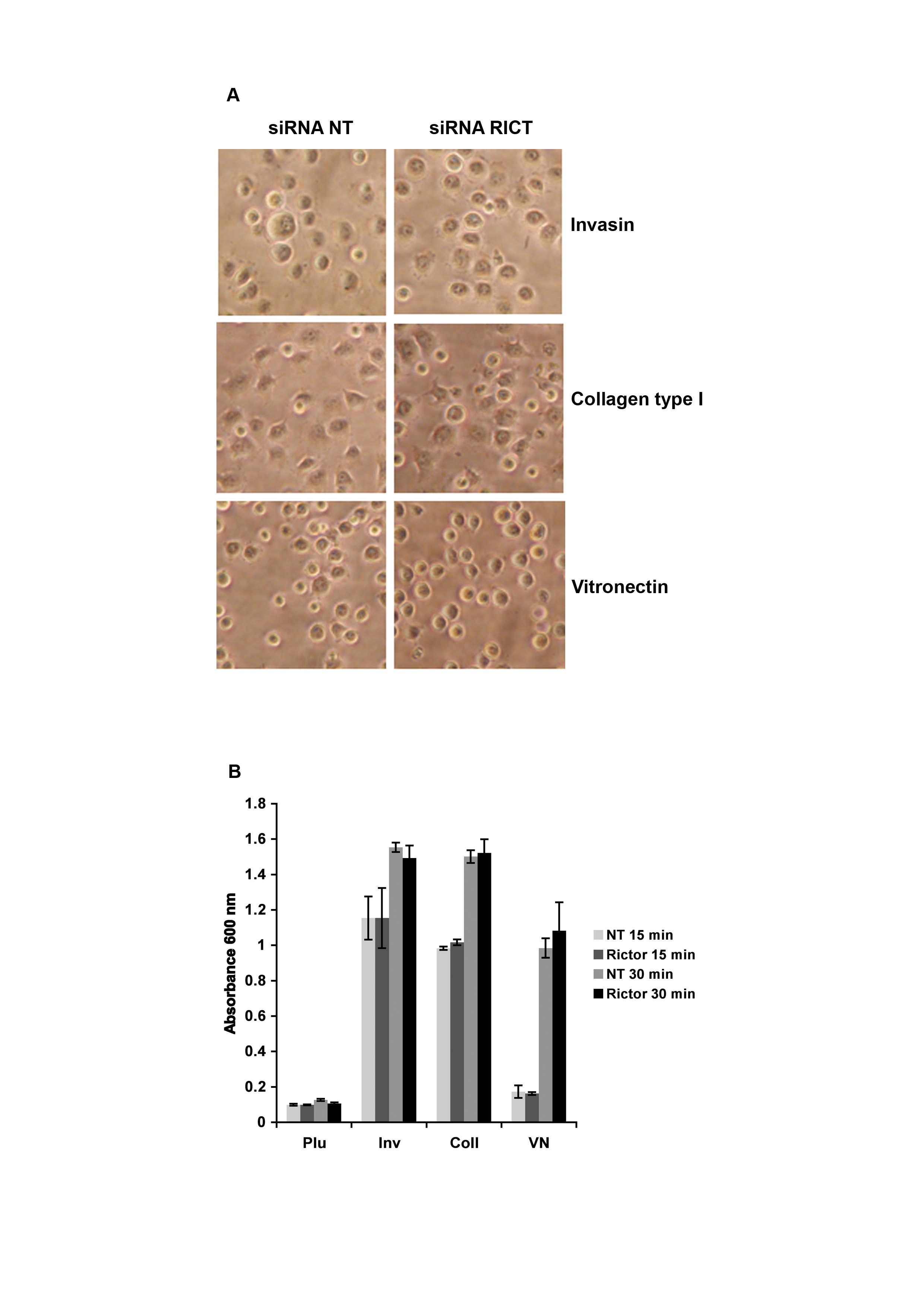

Supplement: Figure S1 — (A) Morphology of HeLa cells transfected with either non-target or RICTOR siRNA after 30 min of spreading on the indicated substrates at 37°C. (B) Attachment assay with HeLa cells transfected with either non-target or RICTOR siRNA. The cells were essentially treated as described under the adhesion assay heading in the Materials and Methods section. 20,000 serum-starved cells per well were seeded in invasin-, collagen-, vitronectin- or Pluronic-coated wells of a 96-well plate and allowed to attach for 15 or 30 min at 37°C. Non-attached cells were removed and the remaining cells were fixed with 96% ethanol for 10 min. Cells were stained for 20 min with 0.1% crystal violet and solubilised in 0.5% SDS. Absorbance was measured at 600 nm. (TIF) [file pone.0032081.s001.tif]

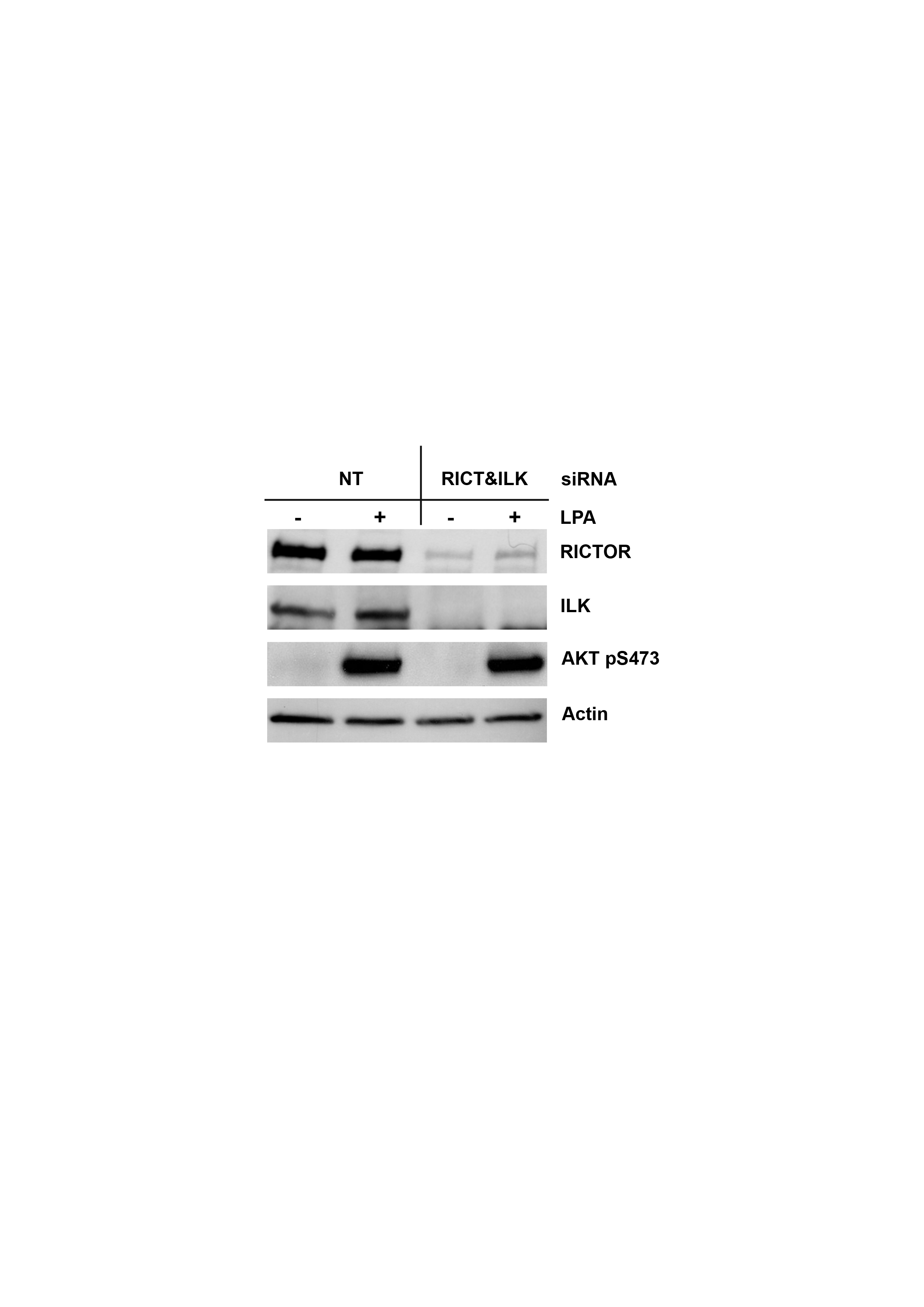

Supplement: Figure S2 — Simultaneous knockdown of RICTOR and ILK does not reduce LPA-mediated AKT Ser473 phosphorylation in HeLa cells. The cells were transfected with RICTOR- and ILK-directed siRNAs simultaneously or with non-target control siRNA and then stimulated with LPA (10 µM) for 20 min. Knockdown efficiency of RICTOR and ILK, and the phosphorylation status of AKT at Ser473 were analysed by western blotting. (TIF) [file pone.0032081.s002.tif]

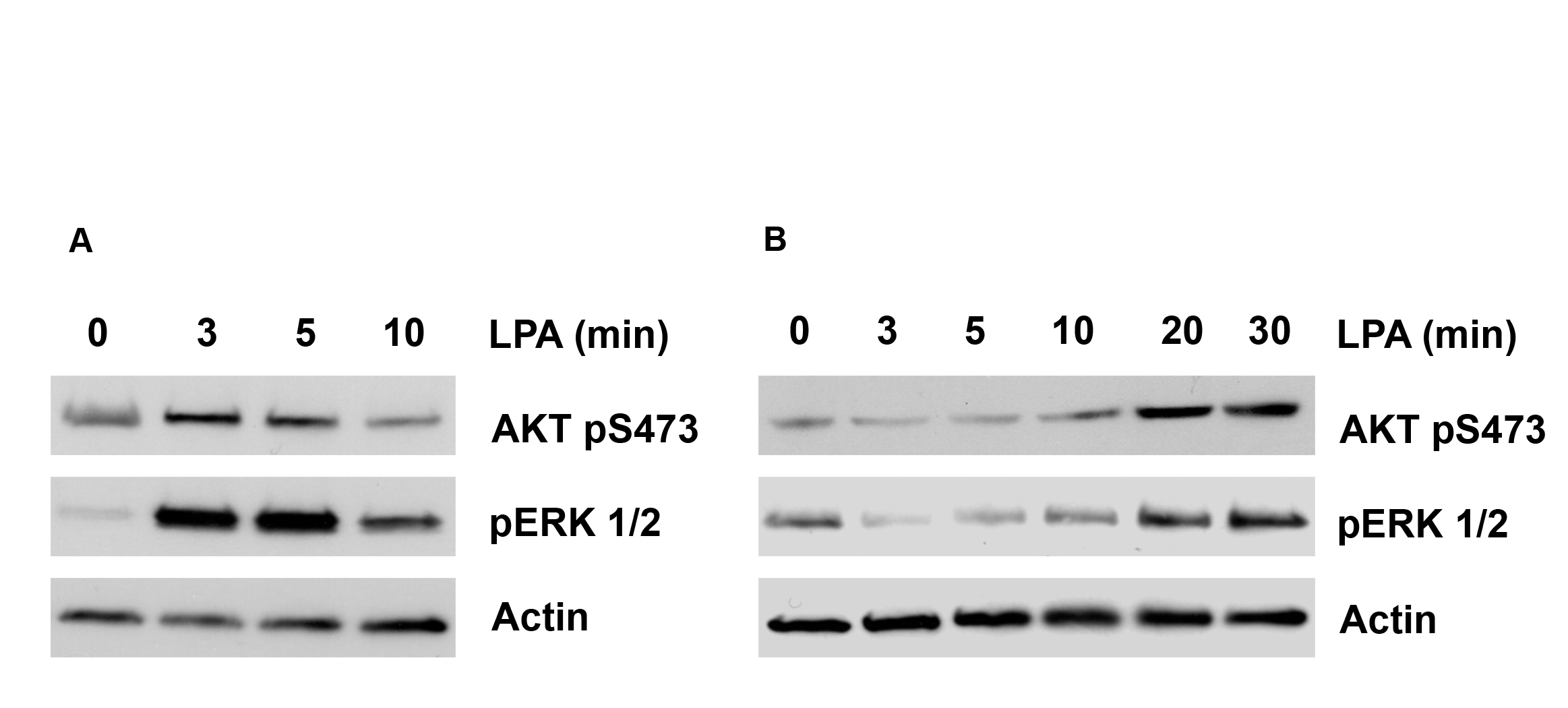

Supplement: Figure S3 — The kinetics of LPA-induced AKT Ser473 phosphorylation are different in HeLa and MCF7 cells (A) HeLa cells were serum-starved and stimulated with LPA (10 µM) for different time periods as indicated. (B) Serum-starved MCF7 cells were stimulated with LPA (5 µM). (TIF) [file pone.0032081.s003.tif]

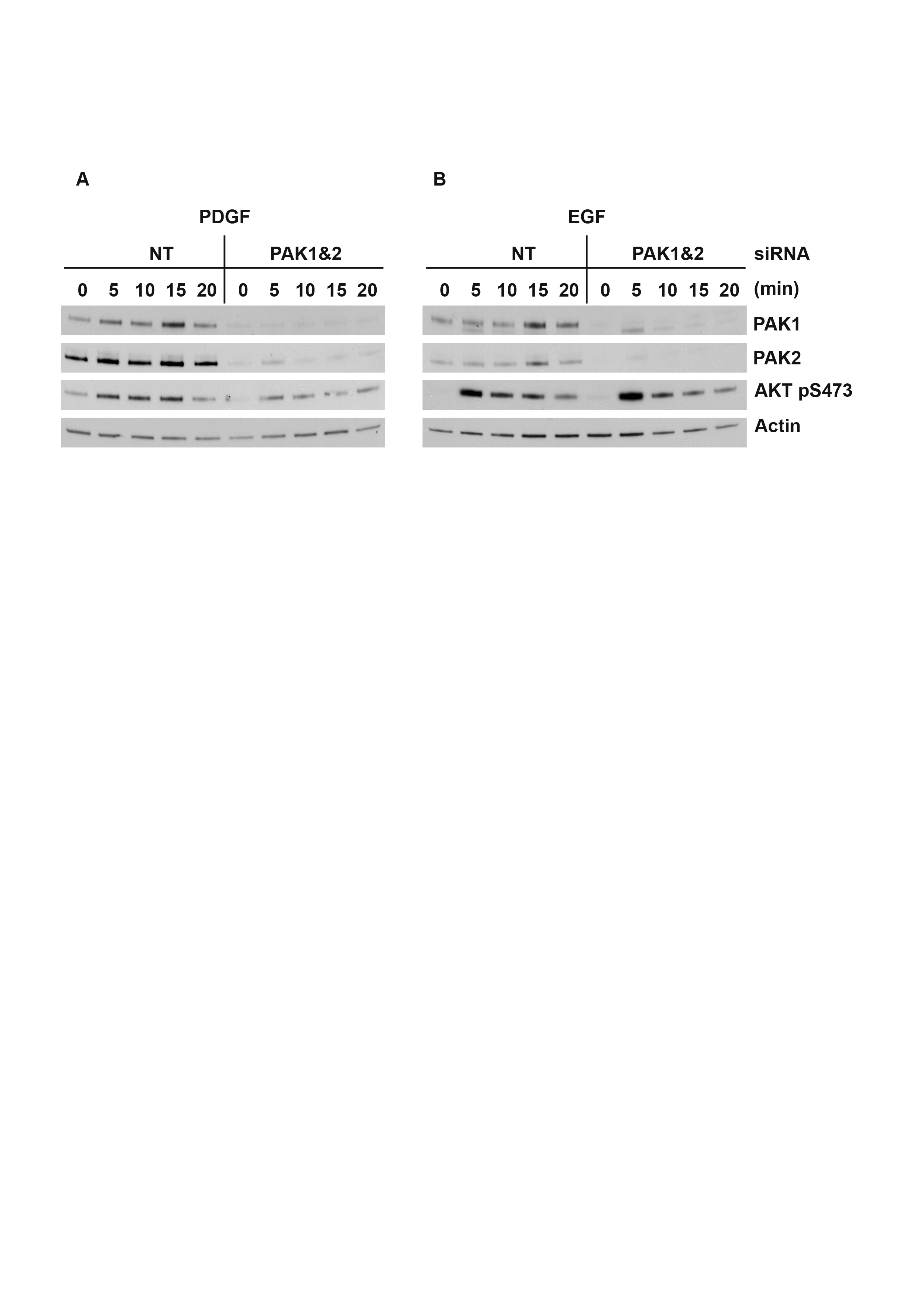

Supplement: Figure S4 — PAK knockdown inhibits PDGF-mediated AKT pSer473, but does not affect kinetics of EGF-induced AKT pSer473. MCF7 cells were transfected simultaneously with PAK1- and PAK2-directed siRNAs or non-target siRNA, grown in complete culture medium for 48 h, serum-starved for 24 h and then stimulated with 20 ng/ml PDGF-BB (A) or 20 ng/ml EGF (B) for the indicated time periods. The efficiency of PAK1 and PAK2 knockdown and the effect of PAK suppression on AKT pSer473 were analysed by western blot. (TIF) [file pone.0032081.s004.tif]
